# Dark Energy with Phantom Crossing and the *H*_0_ Tension

**DOI:** 10.3390/e23040404

**Published:** 2021-03-29

**Authors:** Eleonora Di Valentino, Ankan Mukherjee, Anjan A. Sen

**Affiliations:** 1 Institute for Particle Physics Phenomenology, Department of Physics, Durham University, Durham DH1 3LE, UK; 2 Centre for Theoretical Physics, Jamia Millia Islamia, New Delhi 110025, India; ankan.ju@gmail.com (A.M.); aasen@jmi.ac.in (A.A.S.); 3 Department of Physics, Bangabasi College, Kolkata 700009, India; 4 School of Arts and Sciences, Ahmedabad University, Ahmedabad 380009, India

**Keywords:** dark energy, cosmic microwave background, Hubble tension

## Abstract

We investigate the possibility of phantom crossing in the dark energy sector and the solution for the Hubble tension between early and late universe observations. We use robust combinations of different cosmological observations, namely the Cosmic Microwave Background (CMB), local measurement of Hubble constant ($H_{0}$), Baryon Acoustic Oscillation (BAO) and SnIa for this purpose. For a combination of CMB+BAO data that is related to early universe physics, phantom crossing in the dark energy sector was confirmed at a 95% confidence level and we obtained the constraint $H_{0}=71.0_{-3.8}^{+2.9}$ km/s/Mpc at a 68% confidence level, which is in perfect agreement with the local measurement by Riess et al. We show that constraints from different combinations of data are consistent with each other and all of them are consistent with phantom crossing in the dark energy sector. For the combination of all data considered, we obtained the constraint $H_{0}=70.25\pm 0.78$ km/s/Mpc at a 68% confidence level and the phantom crossing happening at the scale factor $a_{m}=0.851_{-0.031}^{+0.048}$ at a 68% confidence level.

## 1. Introduction

The observed phenomenon of cosmic acceleration [1,2] brought revolutionary change in our understanding of the cosmos. To explain the alleged accelerated expansion within the regime of General Relativity, it is essential to introduce some unknown source in the energy budget of the universe. This exotic source of energy is dubbed as *dark energy*. Different prescriptions from different branches of theoretical physics regarding the physical entity of dark energy are available in the literature (see [3] and references therein). The energy density of a vacuum [4,5,6], scalar fields’ energy density [7], or some unknown fluid [8,9] can be candidates for dark energy. However, none of these are beyond ambiguity. The unprecedented technical developments in cosmological observations in the recent years, like the observation of Cosmic Microwave Background (CMB) by Planck [10], the extended Supernova Cosmology Project [11], the observation of baryon distribution in the universe by the Baryon Oscillation Spectroscopic Survey (BOSS) [12], multi-wavelength observation of the large-scale structure of the universe by the Sloan Digital Sky Survey (SDSS) [13], etc., have ensured very precise constraints on cosmological models.

Depending on the nature of the dark energy equation of state, the time varying dark energy models are classified into two sections, namely phantom dark energy ($w_{de}<-1$) and non-phantom dark energy ($w_{de}>-1$). The phantom barrier is delineated by $w_{de}=-1$, which represents the cosmological constant or the vacuum dark energy. The prime motivation of the present work is to check whether cosmological observations allow a dark energy to have a transition from phantom to non-phantom or vice versa. The theoretical background of phantom crossing dark energy are discussed in [14,15,16,17,18,19,20], in the composite scalar field model in [21,22,23], and in the context of Horndeski’s Theory [24,25]. Some recent studies regarding the observational aspects of phantom crossing dark energy are referred to in [26,27,28,29]. A recent model independent reconstruction of a dark energy equation state by Zhao et al. [30,31] shows that a combination of cosmological data including CMB data from Planck observations points towards possible phantom crossing in a dark energy equation of state. Similar results have been obtained by Capozziello et al. [32] with only low-redshift data. Moreover, reconstruction procedures for the dark energy density $\rho_{de}\left(z\right)$ [29] as well as Hubble parameter H(z) [33] also exhibited phantom crossing in the dark energy sector.

Another serious issue of dark energy reconstruction is the disagreement of the local measurement of the Hubble parameter with the value estimated from the CMB. The local measurements suggest a higher value of the present Hubble parameter ($H_{0}$) compared to the value estimated for the standard model composed by a cosmological constant with cold dark matter (ΛCDM) from the CMB likelihood. The latest measurement of $H_{0}$, reported by the SH0ES collaboration, is $H_{0}=74.03\pm 1.42$ km/s/Mpc at a 68% confidence level (CL) [34] and the value estimated by Planck for ΛCDM is $H_{0}=67.27\pm 0.60$ km/s/Mpc at a 68% CL [35]. The tension is now at a 4.4σ level. There are many attempts to alleviate the issue in the literature (see for an incomplete list of works Refs. [36,37,38,39,40,41,42,43,44,45,46,47,48,49,50,51,52,53,54,55,56,57,58,59,60,61,62,63,64,65,66,67,68,69,70,71,72,73,74,75,76,77,78] and the recent overview in [79,80]). It has been recently discussed [81,82] that a transition in absolute magnitude $M_{B}$ for SnIa can also explain the apparent tension between the local and CMB measurements of the Hubble parameter $H_{0}$. Such variation in $M_{B}$ in SnIa can be related to the apparent variation of the normalized Newtonian constant $\mu =G_{eff}/G_{N}$.

An important aspect of the present reconstruction is, therefore, to investigate whether a phantom crossing in dark energy evolution can alleviate the present Hubble tension. The present reconstruction is purely phenomenological based on the parametrization of the dark energy density. There is no assumption about the physical entity of dark energy from any theoretical background apart from that it has a phantom crossing at some stage during its evolution. The dark energy density is parametrized using a Taylor series expansion truncated at a certain order. The coefficients of the series expansion are constrained using observational data with a statistical approach. We have assumed that the components in the energy budget, namely the matter, dark energy and radiation, are independently conserved. In the following sections, we discuss the present reconstruction, the observational constraints and finally conclude with overall remarks on the results.

## 2. Reconstruction of the Model

One can parametrize the phantom crossing behavior in the dark energy either through its equation of state $w_{DE}\left(z\right)$ or directly through its energy density $\rho_{DE}\left(z\right)$. On the one hand, different observables are directly related to the Hubble parameter H(z) rather than the equation of state of the dark energy fluid. If one parametrizes dark energy with $w_{DE}\left(z\right)$, the dark energy contribution in H(z) involves the integration of $w_{DE}\left(z\right)$ over the redshift interval, whereas parametrizing dark energy with $\rho_{DE}\left(z\right)$ contributes directly to H(z). Hence $\rho_{DE}\left(z\right)$ is the simpler and more direct way to parametrize the dark energy contribution in H(z). Hence we chose $\rho_{DE}\left(z\right)$ to model the dark energy behavior.

Let us write the energy conservation equation for the dark energy fluid: $\frac{d\rho_{DE}}{da}=-\frac{3}{a}\left(1+w_{DE}\right)\rho_{DE}$. It is straightforward to see that for $(1+w_{DE})>0$ (non-phantom models), $\rho_{DE}$ decreases with the scale factor, whereas for $(1+w_{DE})<0$ (phantom models) $\rho_{DE}$ increases with the scale factor. For $w_{DE}=-1$, $\rho_{DE}$ is constant and that is the "Cosmological Constant". Hence, for any phantom crossing, dark energy density should pass through an extremum at some redshift $a=a_{m}$ where $\frac{d\rho_{DE}}{da}$ changes its sign. We perform a Taylor series expansion of $\rho_{DE}$ around this extremum at $a=a_{m}$:

(1) $$\begin{array}{c} \rho_{DE}\left(a\right)=\rho_{0}+\rho_{2}{\left(a-a_{m}\right)}^{2}+\rho_{3}{\left(a-a_{m}\right)}^{3}\\ =\rho_{0}\left[1+\alpha {\left(a-a_{m}\right)}^{2}+\beta {\left(a-a_{m}\right)}^{3}\right]. \end{array}$$

Here we normalize the present day scale factor $a_{0}=1$. As we have assumed that $\rho_{DE}$ has an extrema at $a_{m}$, we have ignored the first order derivative term in the Taylor expansion. We also restricted ourselves up to the third order in the Taylor expansion. Allowing higher-order terms will involve more parameters in the model that may not be tightly constrained with present data. One should also note that there can be a second extrema in $\rho_{DE}$ depending on the values of α and β. With this, the Hubble parameter can be written as:

(2) $$3H^{2}+3\frac{k}{a^{2}}=8\pi G\left[\rho_{m}+\rho_{\gamma }+\rho_{DE}\right].$$

Finally we will have:

(3) $$\begin{array}{c} H^{2}\left(a\right)/H_{0}^{2}=\Omega_{m0}a^{-3}+\Omega_{k0}a^{-2}+\Omega_{\gamma 0}a^{-4}+\\ +\left(\frac{1-\Omega_{m0}-\Omega_{k0}-\Omega_{\gamma 0}}{1+\alpha {\left(1-a_{m}\right)}^{2}+\beta {\left(1-a_{m}\right)}^{3}}\right)\left[1+\alpha {\left(a-a_{m}\right)}^{2}+\beta {\left(a-a_{m}\right)}^{3}\right], \end{array}$$

and the dark energy equation of state:

(4) $$w_{DE}\left(a\right)=-1-\frac{a[2\alpha \left(a-a_{m}\right)+3\beta {\left(a-a_{m}\right)}^{2}]}{3[1+\alpha {\left(a-a_{m}\right)}^{2}+\beta {\left(a-a_{m}\right)}^{3}]}.$$

One can easily rewrite the above expression for $w_{DE}\left(a\right)$ to show that it represents a Pade series of order (3,3), which has a better convergence radius. Additionally, for early times (a→0), the equation of state $w_{DE}\to -1$ shows the Cosmological Constant behavior for the dark energy. This confirms that the dark energy equation of state is well behaved at an early time without any convergence issues. It is also not difficult to verify that adding higher-order terms in $\rho_{DE}$ does not change the $w_{DE}\to -1$ behavior at an early time. We should add that the different terms in the expression for $\rho_{DE}$ can be generated by non-canonical scalar fields with Lagrangian $L\propto -X^{n/(2(3+n))}$ with different values of *n*, as shown in [83].

A set of model parameters $\left(\alpha ,\beta ,a_{m}\right)$ are introduced through the present reconstruction. Clearly the present model mimics the ΛCDM for α=β=0. The $a_{m}$ is the scale factor, where the $\rho_{DE}$ has an extrema. If $a_{m}$ is constrained to be $a_{m}<1$ (we fix $a_{0}=1$ for the present day scale factor), it is a signature of transition in the nature of dark energy. In our subsequent analysis, we assume a spatially flat universe, i.e., $\Omega_{k0}=0$. We allow the dark energy density $\rho_{DE}$ to become negative, as considered by other works (see for example [29,33,84,85]).

## 3. Methodology

In order to constrain the Dark Energy models’ parameters, we make use of some of the most recent cosmological measurements available. These will be:

- **CMB**: we consider the temperature and polarization CMB angular power spectra of the Planck legacy release of 2018 *plikTTTEEE+lowl+lowE* [35,86] as a baseline (Note that there is an alternative likelihood for the Planck data, CamSpec [87], but they are consistent, as stated clearly from the Planck collaboration).
- **R19**: we adopt the gaussian prior $H_{0}=74.03\pm 1.42$ km/s/Mpc at a 68% CL on the Hubble constant as measured by the SH0ES collaboration in [34].
- **BAO**: we add the Baryon Acoustic Oscillation (BAO) measurements 6dFGS [88], SDSS MGS [89], and BOSS DR12 [90], as adopted by the Planck collaboration in [35] (Note that there is an updated version of the BAO data [91], but we prefer to keep the combination used in the literature, for a better comparison).
- **Pantheon**: we make use of the luminosity distance data of 1048 type Ia Supernovae from the Pantheon catalog [92].
- **Lensing**: we consider the 2018 CMB lensing reconstruction power spectrum data, obtained with a CMB trispectrum analysis in [93].

We adopt as a baseline a nine-dimensional parameter space, i.e., we vary the following cosmological parameters: the baryon energy density $\Omega_{b}h^{2}$, the cold dark matter energy density $\Omega_{c}h^{2}$, the ratio of the sound horizon at decoupling to the angular diameter distance to last scattering $\theta_{MC}$, the optical depth to reionization τ, the amplitude and the spectral index of the primordial scalar perturbations $A_{s}$ and $n_{s}$, and, finally, the three parameters assumed in our expansion of the $\rho_{DE}$ in Equation (1), i.e., α, β and $a_{m}$. We impose flat uniform priors on these parameters, as reported in Table 1.

To analyze the data and extract the constraints on these cosmological parameters, we used our modified version of the publicly available Monte Carlo Markov Chain package CosmoMC [94]. This is equipped with a convergence diagnostic based on the Gelman and Rubin statistic [95], assuming R−1<0.02, and implements an efficient sampling of the posterior distribution using the fast/slow parameter decorrelations [96]. CosmoMC includes support for the 2018 Planck data release [86] (see http://cosmologist.info/cosmomc/). Finally, since for point α=β=0, the present model becomes the ΛCDM one, as was already mentioned before, and the likelihood has a singular nature as $a_{m}$ becomes redundant in this case, we switch back to the unmodified CosmoMC code for the analysis of this point, to avoid problems.

## 4. Observational Constraints

**Figure 1:**
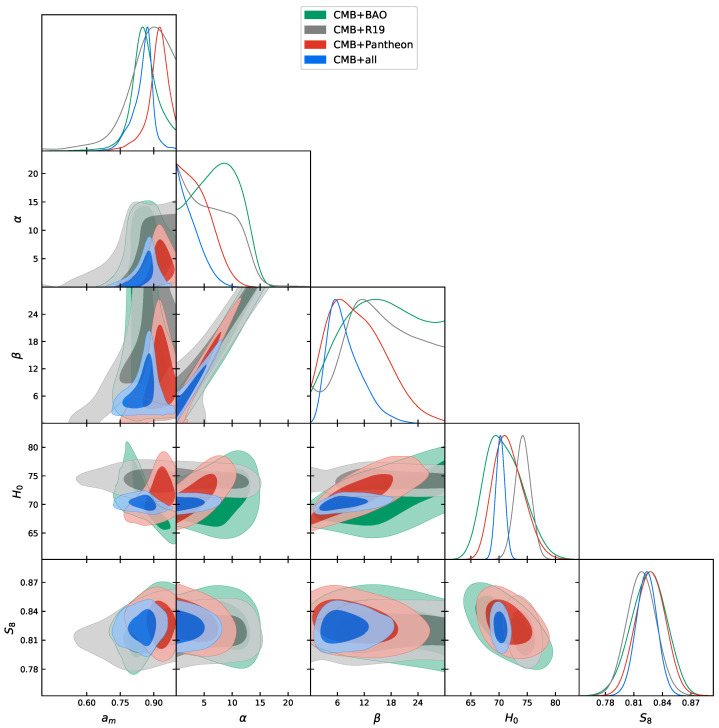
Triangular plot showing 2D and 1D posterior distributions of some interesting parameters considered in this work. Planck+all refers to Planck+lensing+BAO+R19+Pantheon. CMB: Cosmic Microwave Background; BAO: Baryon Acoustic Oscillation.

**Figure 2:**
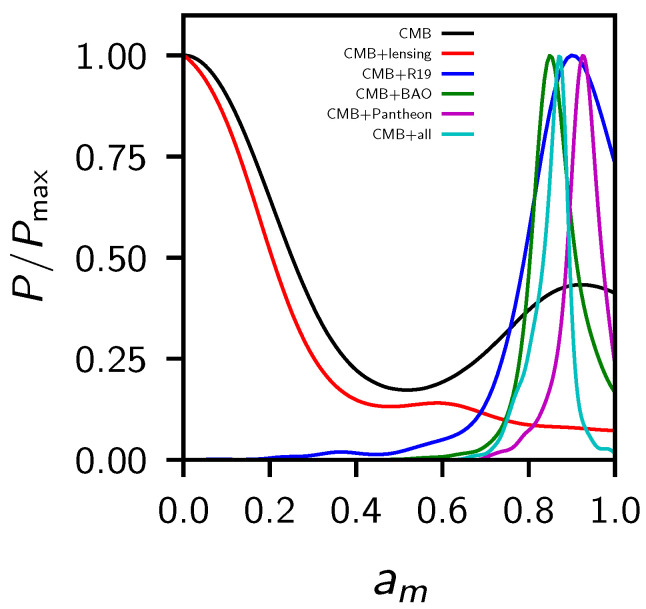
1D posterior distribution of $a_{m}$ for the different dataset combinations explored in this work. CMB+all refers to Planck+lensing+BAO+R19+Pantheon.

In Table 2 we show the constraints at a 68% CL for the cosmological parameters explored in this paper, for different dataset combinations. In Figure 1 we show instead the 2D contour plots and 1D posterior distribution on some of the most interesting parameters. We are not showing the CMB-only constraints because they are bimodal in $a_{m}$, i.e., CMB alone is not able to distinguish which is its best value in fitting the data, but we need additional probes to break the degeneracy. Eventually, we found that CMB+lensing prefers one of the two peaks, while all the other combinations (+BAO, +Pantheon and +R19) prefer the other peak (see Figure 2). Finally, in Table 3 we compare the $\chi_{bf}^{2}$ of the best fit of the data for the standard ΛCDM model and the phantom crossing. We can see that in all the combinations of data considered here, the phantom crossing model improved the $\Delta \chi^{2}$ with respect to the standard model.

Comparing the constraints on the cosmological parameters reported in Table 2 for our scenario with those reported by the Planck collaboration in [35] for a *w*CDM model, we can see that they are completely in agreement for the CMB+lensing dataset combination (second column). This happens because the scale factor of the transition $a_{m}$ is consistent with zero, so in agreement with a phantom dark energy as preferred by Planck in the *w*CDM model. Moreover, both α and β are consistent with 0, i.e., a cosmological constant, within one standard deviation. For CMB+lensing we found that the Hubble constant parameter is almost unconstrained ($H_{0}>75.4$ km/s/Mpc at a 95% CL), and $S_{8}=0.752_{-0.025}^{+0.009}$ at a 68% CL is completely in agreement within one standard deviation with the combination of the cosmic shear data KiDS+VIKING-450+DES-Y1 [97], whereas the tension on $S_{8}$ is at 3.2σ in a ΛCDM context.

Since the CMB and R19 are in agreement now within two standard deviations, we can combine them safely together. The results we obtained for the joint analysis CMB+R19 are reported in the third column of Table 2. Here we see that while α is still consistent with zero within 1σ, we now have β=16.0±7.5 at a 68% CL and $a_{m}>0.830$ at a 68% CL, i.e., consistent with 1.

**Figure 3:**
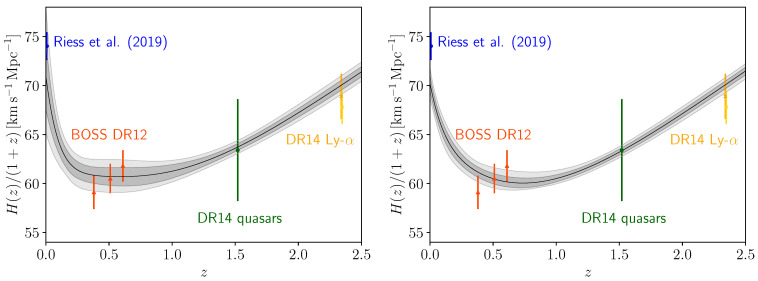
Behavior of H(z)/(1+z) for the combinations CMB+BAO (on the **left**) and CMB+all (on the **right**). Observational data points of local measurement of $H_{0}$ by Riess et al. [34], BOSS DR12 [90], BOSS DR14 quasars [98], and BOSS DR14 Ly-α [99,100] are also shown.

An interesting result is the one obtained combining CMB and BAO together, which is shown in the forth column of Table 2. Here we can see that contrary to many other cosmological scenarios, including a ΛCDM model of which our parametrization is an extension, CMB+BAO gave $H_{0}=71.0_{-3.8}^{+2.9}$ km/s/Mpc at a 68% CL. This large Hubble constant value is now perfectly consistent within one standard deviation with the R19 measurement, while all other cosmological parameters are almost unchanged if compared with a *w*CDM scenario for the same CMB+BAO data combination. This increase of the $H_{0}$ parameter is due to its positive correlation with α and β, and negative correlation with $a_{m}$, as can be seen in Figure 1. For the CMB+BAO case we have in fact an indication that all these three parameters are different from the expected values at more than 1σ. In particular we find, at a 68% CL, $a_{m}=0.859\pm 0.064$, α=7.3±3.9 and β=16.1±7.8. Therefore, in this case there is an indication at more than 2σ for a transition in the dark energy density. The constraint on the present day equation of state $w_{DE}\left(z=0\right)$ is $-1.61_{-0.91}^{+0.60}$ at a 95% CL, ruling out the cosmological constant at about 2σ. Given that both CMB and BAO are related to early universe physics, this shows that a phantom crossing in dark energy sectors alleviates the tension between the early and late universe determinations of the parameter $H_{0}$. In the left panel of Figure 3, we show the behavior of the expansion rate of the universe for this dataset combination. We can see an excellent agreement with all the latest measurements. This agreement finds confirmation in the $\chi_{bf}^{2}$ (see Table 3), where we show that the phantom crossing model improved the $\Delta \chi^{2}$ with respect to the standard ΛCDM model, not only for the Planck+BAO combination, but also for the BAO data alone.

The same interesting larger value of the Hubble constant persists even if we combine CMB and Pantheon data. In this case, as we show in the fifth column of Table 2, $H_{0}=71.7_{-3.1}^{+2.2}$ km/s/Mpc at a 68% CL, i.e., consistent with R19. As can be seen in Figure 1, it is the positive correlation between $H_{0}$ and α and β that shifts the Hubble constant towards higher values, whereas, on the contrary with respect to the CMB+BAO combination, in this case there is a positive correlation also between $H_{0}$ and $a_{m}$. For the dark energy parameters of our model we found for CMB+Pantheon, at a 68% CL, $a_{m}=0.917_{-0.029}^{+0.054}$ and $\beta =10.6_{-7.9}^{+4.4}$, i.e., different from the expected value in a ΛCDM model at more than 1σ, while α<5.10 is consistent with zero.

Given a preference for all data combinations of a large $H_{0}$, we can conclude that this indication is robust irrespective to the combination of data analyzed here. For this reason we combine them all together because they are no more in tension. In fact, even the Planck+lensing dataset combination resulted in $H_{0}>75.4$ km/s/Mpc at a 95% CL, i.e., in perfect agreement with R19. The joint result, i.e., CMB+lensing+BAO+Pantheon+R19, is displayed in the last column of Table 2, where we see $H_{0}=70.25\pm 0.78$ km/s/Mpc at a 68% CL, reducing the tension with R19 at 2.3 standard deviations. In the right panel of Figure 3, we show the behavior of the expansion rate of the universe for this combination. Also in this case, we can see a good agreement with all of the latest measurements of BAO. However, even if for this dataset combination we have a slightly lower $S_{8}=0.823\pm 0.011$ at a 68% CL, the tension with the cosmic shear data KiDS+VIKING-450+DES-Y1 [97] is still at 3.1σ. For the joint case we found, at a 68% CL, $a_{m}=0.851_{-0.031}^{+0.048}$ and $\beta =7.7_{-4.7}^{+2.2}$, i.e., they are different from the expected value in a ΛCDM scenario at more than 2σ because they are highly non-gaussian, while α<3.32 at a 68% CL is consistent with zero. Therefore, a robust indication at more than 2σ for a transition in the dark energy density is suggested by the data. The constraint on the present day equation of state $w_{DE}\left(z=0\right)$ is $-1.33_{-0.42}^{+0.31}$ at a 95% CL, ruling out the cosmological constant at more than 2σ. Finally, if we look at the $\chi_{bf}^{2}$ in Table 3, we can see that the Phantom Crossing model improves significantly the total $\Delta \chi^{2}$ we had for the standard ΛCDM model.

From the constraints on $r_{d}$ in Table 3 for different data combinations, especially for the CMB+BAO combination, we can say that our results agree with BAO data, as well as a larger $H_{0}$ value, even if we do not change $r_{d}$ as constrained by Planck for ΛCDM. This may be due to non-monotonic dark energy evolution in late time.

It is also not difficult to check that $\rho_{DE}\left(z\right)$ for the constrained parameter space can become negative for some redshifts and this is consistent with earlier results by [29,33,84,85]. A negative $\rho_{DE}\left(z\right)$ at some earlier time may help reduce the Hubble tension.

Additionally, to perform a model comparison, we computed the Bayesian evidence and we show the results in Table 4. This allowed us to quantify which model fits the data better between ΛCDM and phantom crossing. We used the publicly available cosmological code MCEvidence (https://github.com/yabebalFantaye/MCEvidence [101,102]). We assumed that for negative (positive) values of the Bayes factor $\ln B_{ij}$, the ΛCDM (phantom crossing) is the preferred model. To interpret the results, we referred to the revised Jeffreys scale by Kass and Raftery as in Reference [103]. Therefore, we will have for $0\leq |\ln B_{ij}|<1$ weak evidence, for $1\leq |\ln B_{ij}|<3$ definite evidence, for $3\leq |\ln B_{ij}|<5$ strong evidence, and for $|\ln B_{ij}|\geq 5$ very strong evidence for one model versus the second one. Looking at Table 4 we can see that we have very strong evidence for the phantom crossing for CMB+R19, while the ΛCDM model is preferred for all other dataset combinations.

## 5. Conclusions

In this work, we considered a dark energy behavior with phantom crossing and confronted it with different observational data including the latest CMB data from Planck. We did not consider any specific theoretical setup involving fields but rather we approached it in a general way where we assumed that the dark energy density should have an extrema at a particular scale factor $a_{m}$ for phantom crossing. If $a_{m}<1$, this crossing happens before the present day. We Taylor expanded the dark energy density around this extrema and checked whether the observational data are consistent with $a_{m}<1$. We found that a combination of observational data including that from Planck is indeed consistent with $a_{m}<1$, confirming the presence of phantom crossing. Moreover, the phantom crossing also helps to alleviate the $H_{0}$ tension between low and high redshift observations. The CMB+BAO combination, which represents early universe physics, gave the constraint $H_{0}=71.0_{-3.8}^{+2.9}$ km/s/Mpc at a 68% CL for the model with phantom crossing, which is fully in agreement with the local measurement of $H_{0}$ by R19. Moreover, constraints on different parameters including $H_{0}$ for different combinations of data were found to be consistent with each other, which allowed us to combine all the data. For the combination of all data, the phantom crossing was observed at more than 2σ and the constraint on $H_{0}$ was $H_{0}=70.25\pm 0.78$ km/s/Mpc at a 68% CL, which is in tension with R19 at 2.3 standard deviations—much lower than the present tension with ΛCDM and many other dark energy models, suggesting a substantial alleviation of the Hubble tension with phantom crossing. Finally, as seen in Table 3, the phantom crossing model fit better than ΛCDM the full dataset combination, improving the $\chi_{bf}^{2}$.

## Figures and Tables

**Table 1:** Flat priors for the cosmological parameters.

Parameter	Prior
$\Omega_{b}h^{2}$	[0.005,0.1]
$\Omega_{c}h^{2}$	[0.005,0.1]
τ	[0.01,0.8]
$n_{s}$	[0.8,1.2]
$\log[10^{10}A_{s}]$	[1.6,3.9]
$100\theta_{MC}$	[0.5,10]
α	[0,30]
β	[0,30]
$a_{m}$	[0,1]

**Table 2:** 68% confidence level (CL) constraints on the cosmological parameters for the different dataset combinations explored in this work. CMB+all refers to Planck+lensing+BAO+R19+Pantheon.

Parameters	CMB+Lensing	CMB+R19	CMB+BAO	CMB+Pantheon	CMB+All
$a_{m}$	<0.276	>0.830	0.859±0.064	$0.917_{-0.029}^{+0.054}$	$0.851_{-0.031}^{+0.048}$
α	<17.7	<8.62	7.3±3.9	<5.10	<3.32
β	<16.7	16.0±7.5	16.1±7.8	$10.6_{-7.9}^{+4.4}$	$7.7_{-4.7}^{+2.2}$
$\Omega_{c}h^{2}$	0.1194±0.0014	0.1196±0.0014	0.1201±0.0013	0.1198±0.0014	0.1198±0.0011
$\Omega_{b}h^{2}$	0.02243±0.00014	0.02243±0.00016	0.02238±0.00014	0.02240±0.00015	0.02240±0.00014
$100\theta_{MC}$	1.04097±0.00031	1.04096±0.00032	1.04092±0.00030	1.04095±0.00032	1.04093±0.00030
τ	0.0521±0.0076	0.0532±0.0080	$0.0539_{-0.0080}^{+0.0070}$	0.0529±0.0076	0.0521±0.0075
$n_{s}$	0.9667±0.0042	0.9665±0.0045	0.9652±0.0043	0.9659±0.0045	0.9655±0.0038
$\ln(10^{10}A_{s})$	3.038±0.015	3.041±0.016	3.044±0.016	3.041±0.016	3.039±0.015
$H_{0}\left[\mathrm{km}/s/\mathrm{Mpc}\right]$	>92.8	74.2±1.4	$71.0_{-3.8}^{+2.9}$	$71.7_{-3.1}^{+2.2}$	70.25±0.78
$\sigma_{8}$	$1.012_{-0.009}^{+0.051}$	0.881±0.018	$0.848_{-0.034}^{+0.027}$	$0.860_{-0.033}^{+0.026}$	0.838±0.011
$S_{8}$	$0.752_{-0.025}^{+0.009}$	0.818±0.016	0.826±0.019	0.828±0.016	0.823±0.011
$r_{\mathrm{drag}}$	$147.19_{-0.26}^{+0.28}$	147.14±0.30	147.06±0.29	147.10±0.30	147.10±0.25

**Table 3:** $\chi_{\mathrm{bf}}^{2}$s comparison between ΛCDM and Phantom Crossing for the different dataset combinations explored in this work. CMB+all refers to Planck+lensing+BAO+R19+Pantheon.

Λ **CDM**	**CMB+Lensing**	**CMB+R19**	**CMB+BAO**	**CMB+Pantheon**	**CMB+All**
$\chi_{bf,\mathrm{tot}}^{2}$	2782.040	2791.838	2779.712	3807.500	3840.406
$\chi_{bf,\mathrm{CMB}}^{2}$	2778.122	2768.113	2770.060	2767.697	2779.508
$\chi_{bf,\mathrm{lensing}}^{2}$	8.981	−	−	−	9.510
$\chi_{bf,R19}^{2}$	−	18.117	−	−	16.414
$\chi_{bf,\mathrm{BAO}}^{2}$	−	−	6.514	−	5.271
$\chi_{bf,\mathrm{Pantheon}}^{2}$	−	−	−	1035.268	1034.768
**Phantom Crossing**	**CMB+Lensing**	**CMB+R19**	**CMB+BAO**	**CMB+Pantheon**	**CMB+All**
$\chi_{bf,\mathrm{tot}}^{2}$	2776.610	2765.556	2775.204	3805.278	3828.424
$\chi_{bf,\mathrm{CMB}}^{2}$	2770.124	2762.965	2763.945	2765.943	2775.585
$\chi_{bf,\mathrm{lensing}}^{2}$	8.145	−	−	−	8.702
$\chi_{bf,R19}^{2}$	−	0.307	−	−	8.275
$\chi_{bf,\mathrm{BAO}}^{2}$	−	−	5.321	−	5.702
$\chi_{bf,\mathrm{Pantheon}}^{2}$	−	−	−	1036.603	1035.971

**Table 4:** The table shows the values of $\ln B_{ij}$ calculated for the phantom crossing model with respect to the ΛCDM scenario. The negative value in $\ln B_{ij}$ indicates that there is a preference for ΛCDM against the phantom crossing model, while a positive value indicates a preference for phantom crossing.

Data	$\ln B_{\mathrm{ij}}$
CMB	0.30
CMB+lensing	0.13
CMB+R19	6.91
CMB+BAO	−2.29
CMB+Pantheon	−4.46
CMB+all	−1.75

## Data Availability

All the datasets used are publicly available and a description can be found in the methodology section.
